# Determination of Organochlorines in Soil of a Suburban Area of São Paulo Brazil

**DOI:** 10.3390/ijerph17165666

**Published:** 2020-08-05

**Authors:** Justine P. R. O. Varca, Elâine A. J. Martins, Gustavo H. C. Varca, Renato L. Romano, Daniel T. Lebre, Paulo E. O. Lainetti, José O. V. Bustillos

**Affiliations:** 1 Nuclear and Energy Research Institute, São Paulo 05508-000, Brazil; nanijardim01@gmail.com (E.A.J.M.); varca@usp.br (G.H.C.V.); lainetti@ipen.br (P.E.O.L.); ovega@ipen.br (J.O.V.B.); 2 Applied Mass Spectrometry Center (CEMSA), São Paulo 05508-000, Brazil; renatolahos@yahoo.com.br (R.L.R.); daniel.lebre@cemsalab.com.br (D.T.L.)

**Keywords:** organochlorines, soil, contamination, QuEChERS, validation, GC-ECD, landfill

## Abstract

Technological advances have promoted improvements in several science fields, especially related to environmental and analytical areas with the improvement of detection and development of environmentally friendly extraction techniques. This study applied Quick, Easy, Cheap, Effective, Rugged and Safe method (QuEChERS) for soil extraction and assessed its performance through a validation study using samples from the soil of a contaminated area in Caieiras, SP, Brazil. Nine organochlorine pesticides, including the isomers alpha, beta, gamma and delta- hexachlorocyclohexane; *cis-* and *trans*-heptachlor epoxide; *cis-* and *trans*-chlordane and heptachlor were analyzed by gas chromatography coupled to electron capture detector. The method was validated according to ISO 5725-4 (2020), EURACHEM (2014) and DOQ-CGCRE-008 (2016). The limits of detection and quantification of the method for the nine organochlorines were α-HCH (1.2 and 12.6 µg kg^−1^), β-HCH (1.7 and 12.0 µg kg^−1^), γ-HCH (1.5 and 11.6 µg kg^−1^), δ-HCH (0.8 and 11.6 µg kg^−1^), heptachlor (1.0 and 10.8 µg kg^−1^), *cis*-heptachlor epoxide (0.9 and 11.5 µg kg^−1^), *trans-*heptachlor epoxide (0.9 and 11.5 µg kg^−1^), *cis*-chlordane (0.4 and 7.9 µg kg^−1^) and *trans-*chlordane (0.5 and 10.9 µg kg^−1^), respectively, and all of them were within the maximum limits recommended by the EPA for the compounds α-HCH (86.0 and 360.0 µg kg^−1^), β-HCH (300.0 and 1.3 × 10^3^ µg kg^−1^), γ-HCH (570.0 and 2.5 × 10^3^ µg kg^−1^), δ-HCH (not defined), heptachlor (130.0 and 630.0 µg kg^−1^), *cis-/trans-*heptachlor epoxide (7.0 and 330.0 µg kg^−1^), *cis-/trans*-chlordane (1.77 × 10^3^ and 7.7 × 10^3^ µg kg^−1^) in residential and industrial soil, respectively. Recovery results were between 65% and 105% for almost all compounds, which is an optimum result for multi-residue analytical methods, considering the complexity of the matrix used in the study. Caieiras presented contamination levels of α-HCH in the range of 2.0 to 66.0 µg g^−1^, which was higher than the limits established by EPA, corresponding to 0.077 µg g^−1^ for residential soil and 0.27 µg g^−1^ for industrial soil. According to the validation study, the analytical method proposed was reliable for organochlorine quantification, and the QuEChERS was considered efficient for organochlorine extraction from soil.

## 1. Introduction

The interest for green analysis methods using fewer solvents and chemical reagents has increased, and the environmental impact generated by human activities has been estimated through different techniques. A large number of studies over the world have contributed to advances in this field, and several matrices have been analyzed to quantify the presence of pesticides and industrial byproducts in air, water, soil, food, human blood and tissues [1,2,3,4,5,6,7].

Some efforts have been made for countries from all around the world to develop quantification methods for organochlorine contamination study as well as to estimate their impact on the environment and human health. Many studies related to pesticide quantification in the soil are available in the literature, particularly in the context of soil contamination [8,9,10]. However, there are still several contaminated and not studied areas in Brazil as pointed in the “National Implementation Plan Brazil–Stockholm Convention”, which is a federal document elaborated by the Brazilian Environmental Ministry in attendance to the Stockholm treaty, since Brazil is one of the 113 signatory countries [11]. The Stockholm treaty has historical importance since the document introduced concepts such as ecology and environmental education across the globe and recommended reduction or ban of a class of compounds defined as persistent organic pollutants (POPs) [12]. 

Among these POPs are the isomers alpha (α), beta (β), gamma (γ) and delta (δ) hexachlorocyclohexane (HCH-C_6_Cl_6_), heptachlor (C_10_H_5_Cl_7_), *cis-* and *trans*-heptachlor epoxide (C_10_H_5_Cl_7_), and *cis-* and *trans*-chlordane (C_10_H_6_Cl_8_), which were considered in this study. In 2002, the Environmental Company of São Paulo state (CETESB) produced the first document of contaminated areas in Brazil in which 225 areas were registered accounting environmental passives and in 2003 initiated a partnership with the Health Surveillance Agency from Caieiras, SP, and studied soil and water samples from an illegal HCH landfill. The place is surrounded by a population that has been exposed to this contamination since the deposition. The study found the presence of HCH isomers in soil and water, although the study was not conclusive [13].

The Quick, Easy, Cheap, Effective, Rugged and Safe method (QuEChERS), initially developed by Anastassiades and colleagues, initially for food extraction, has led to excellent technical and economic results [14]. The technique has been applied for pesticide determination in several types of food matrices such as fruits, vegetables, grains, flour, bran and tea [15,16,17,18]. Besides food, the QuEChERS method has been used for pesticide determination in soil and veterinary drug residues in animal tissue [19,20] but still requires more studies to demonstrate its efficacy. The method presents several advantages especially regarding its flexibility to be combined with different analytical techniques, low solvents requirements and suitability for fast processing of many compounds in a single assay. The literature has reported several studies with the QuEChERS method successfully applied in the soil to replace many complicated analytical steps commonly employed in traditional methods, providing high-quality results with high sample throughput [21,22,23]. It is composed of three steps: sample preparation, sample extraction and sample extract clean-up. Many works report the clean-up step depending on the complexity of the matrix [10,21,22,23,24,25,26].

The present study aimed to contribute to the accomplishment of the Stockholm treaty signed by Brazil, considering a large number of not studied landfills in the country and taking into account the soil diversity and the environmental passives available around the world which have never been studied nor monitored. In this sense, real samples were collected in Caieiras landfill, SP, Brazil for initial screening of the area and the determination of the contamination levels of the POPs mentioned previously. The determination and quantification were performed by gas chromatography coupled to an electron capture detector (GC-ECD) and the analytical method was validated following the ISO 5725-4 (2020), EURACHEM (2014) and DOQ-CGCRE-008: 2016 [26,27,28]. 

There are many analytical methods for pesticide determination in soil available in the literature, however, in terms of comparison of results, it is quite complex, because of the different soil properties found in the landfills reported. It is known that the analytical method response is impacted by the matrix effect. In the case of a soil matrix, some properties such as pH, which modulates the compound percolation, granulometry, which reveals the soil profile, and organic content, which modulates compound percolation, directly impact the outcome of the method. In this context, the present study is also relevant for providing soil characterization, which might help other studies. Concerning the contamination results, they were compared with the maximum limits for industrial and domestic soil recommended by the Environmental Protection Agency (EPA) [29] showed in Table 1 and other works involving POPs contamination levels quantified in Brazil and around the world.

## 2. Materials and Methods 

### 2.1. Materials

Individual standard solutions of α, β, γ, δ-HCH, heptachlor, *cis, trans-*heptachlor epoxide, *cis* and *trans-*chlordane, and the reagents magnesium sulfate (MgSO_4_), sodium chloride (NaCl), sodium citrate (Na_3_C_6_H_5_O_7_), sodium hydrogen citrate sesquihydrate (C_6_H_6_Na_2_O_7_·1.5 H_2_O) and Bondesil-PSA were bought from Sigma Aldrich (St. Louis, MO, USA). Solvents such as acetonitrile (ACN) and hexane were purchased from J. T. Baker® (Phillipsburg, NJ, USA). Plastic centrifugal tubes (50 and 30 mL) were purchased from Corning TM (Corning, NY, USA). Polypropylene syringe microfilters (diameter of 0.25 mm and pore size of 0.45 µm) were purchased from Advanced MFS Gebhardt (Sinsheim, Germany).

### 2.2. Apparatus

The method was performed in a GC 17A with a nickel-63 electron capture detector model ECD-2010 Exceed (Shimadzu, Kyoto, Japan). The gas chromatographic system was equipped with a 30 m DB-5 capillary column with stationary phase of trifluoropropyl methyl polysiloxane (J&W Scientific, Folsom, CA, USA) with an internal diameter of 0.25 mm and 0.25 µm film thickness. Helium with a purity of 99.999% (Linde, Brazil) was used as a gas carrier with a column head pressure of 12 p.s.i., split/splitless unit. Chromatographic data were collected and recorded using GC Analyst software (Shimadzu, Kyoto, Japan).

### 2.3. Standard Solutions

The organochlorine pesticide stock standard mixture (100 µg mL^−1^) was made by aliquoting the individual stock solutions of alpha-hexachlorocyclohexane (α-HCH), beta-hexachlorocyclohexane (β-HCH), gamma-hexachlorocyclohexane (γ-HCH), delta-hexachlorocyclohexane (δ-HCH), heptachlor, *cis*-heptachlor epoxide, *trans*-heptachlor epoxide, *cis*-chlordane and *trans*-chlordane (1.0 mg mL^−1^ in hexane). The analytical curve was prepared in two media, in acetonitrile (ACN) and soil matrix, and for that, the standard mixture was diluted in seven points, in the range of 100 to 1160 µg kg^−1^.

### 2.4. Soil Collection and Classification

**Figure 1:**
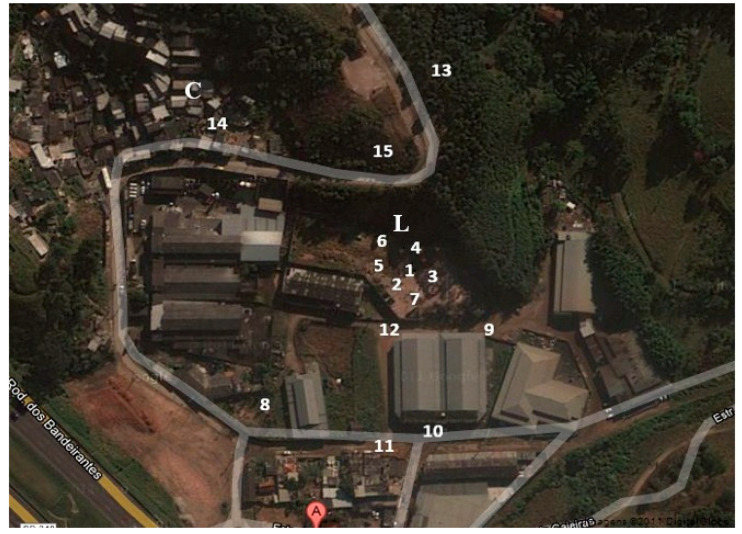
Image of Landfill (L) and the community (C) areas in Caieiras, SP, Brazil, in which each sampling point is shown (Picture adapted from Google Maps).

Fifteen soil samples were collected from an unduly landfill in Caieiras, SP, Brazil, from the surface to 30 cm of depth and appropriately stored according to the protocol described by CETESB [30]. The exact location of the sampling, according to the global positional system (GPS) coordinates is shown in Table 2, the collect region is shown in Figure 1, and the sampling area was around 1200 m^2^. For soil classification and method validation, a pool sample was made by mixing and quartering portions of 100 g from each of the fifteen samples. The soil was classified in terms of pH, granulometry [31] and organic matter content following the Guide of methods for soil analysis of CETESB and the method for determination of organic matter content by burning at 440 °C of Technical Rules Brazilian Association (ABNT NBR 13600–1996) [32].

### 2.5. Extraction

**Figure 2:**
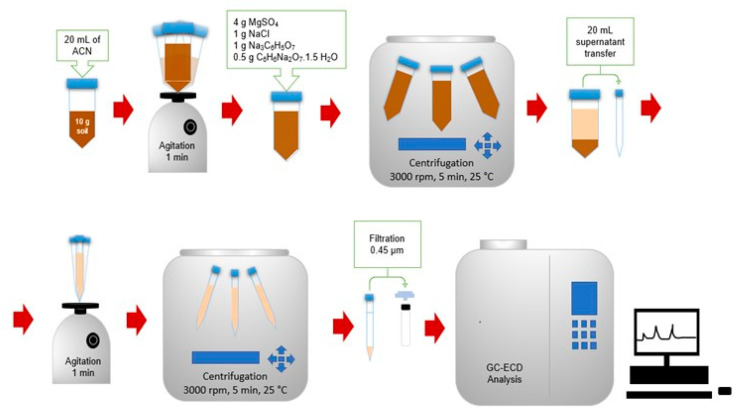
Representative scheme of the analytical method.

The extraction was performed by weighing 10 g of sample in a 50 mL plastic centrifugal tube followed by the addition of 20 mL of ACN and vortexing for 1 min. After that, 4 g of MgSO_4_, 1 g of NaCl, 1 g of Na_3_C_6_H_5_O_7_, 0.5 g of C_6_H_6_Na_2_O_7_·1.5 H_2_O were added to the tubes. The mixture was then vortexed for 1 minute and centrifuged for 5 min at 3000 rpm and 25 °C. Then, an aliquot of the supernatant was transferred to a 30 mL plastic centrifugal tube followed by the clean-up step, which consisted of adding 0.95 g of magnesium sulfate and 0.15 g of Bondesil-PSA according to Prestes and colleagues [33]. The sample was vortexed for 1 min and centrifuged again under the same conditions as mentioned above. Finally, the supernatant was collected and stored at 4 °C for analysis. The extraction method is illustrated in Figure 2.

### 2.6. GC-ECD Technique

The GC-ECD conditions applied were 250 °C for injection temperature, 280 °C for interface temperature, 2.5 kV for voltage detector, helium as the gas carrier (1.1 mL min^−1^) and temperature gradient of 80 to 210 °C at 25 °C min^−1^ and 210 to 290 °C at 35 °C min^−1^. The representative scheme of the analytical method developed in this work is shown in Figure 2.

### 2.7. Quality Assurance

The quality assurance was performed through validation tests based on the criteria established by the European Guide, EURACHEM: 2014 [26], International Standardization Organization (ISO 5725-4:2020) [27] and the national guidance of validation of analytical methods of Metrology, Quality and Technology National Institute (DOQ-CGCRE-008:2016) [28]. The validation parameters included linearity, which was assessed by the linear regression equation and determination coefficients; selectivity and sensitivity, which were evaluated through the comparison between chromatograms of the standard mixture prepared in ACN solvent (0.1 µg mL^−1^) and in soil matrix (100 µg kg^−1^); accuracy, which was assessed by trueness, evaluated by the Z-score test of recovery, and precision, assessed through repeatability, through the analysis of the standard mixture prepared in soil matrix, in three different concentration levels, in seven replicates, in the same day, equipment and operator and calculating the limit of repeatability (r) from the relative standard deviation; reproducibility, considering three concentration levels and seven replicates analyzed in different days and analysts; limits of detection and quantification, determined by analytical curves, in solvent and in soil matrix; and recovery.

Recovery assays were performed by fortifying 10 g of soil sample collected at the same region of the samples analyzed, with a pesticide standard mixture prepared in two concentration levels (1.0 and 2.0 µg g^−1^) for 24 hours, followed by extraction by the QuEChERS method. To compare the area values obtained from the chromatograms, the fortification was performed in the same concentrations as for the soil samples after the extraction procedure. The experiments were made in triplicate, and the extracts were evaluated by GC-ECD. Equation (1) shows the formula used to calculate the recovery, considering that *R* (%) means recovery expressed in percentage, *A*_1_ means fortification before extraction and *A*_2_ fortification after extraction. The blank soil was prepared and extracted by QuEChERS as well as the rest of the samples. All samples were assessed in triplicate.

(1) $$R\;\left(\%\right)=\frac{A_{1}}{A_{2}}100$$

In practical terms, the validation experiment was performed through the analysis of the standard mixture of the nine pesticides previously prepared in both ACN solvent and soil matrix, in seven replicates and seven concentration levels. In compliance with the validation guides, the validation was performed based on intraday and interday assays.

### 2.8. Statistical Analysis

Regression analysis, average, standard deviation, coefficient of variation and analysis of variance (ANOVA) of the data were determined using Microsoft Office Excel 365 (New York, NY, USA).

## 3. Results

### 3.1. Method Development

**Figure 3:**
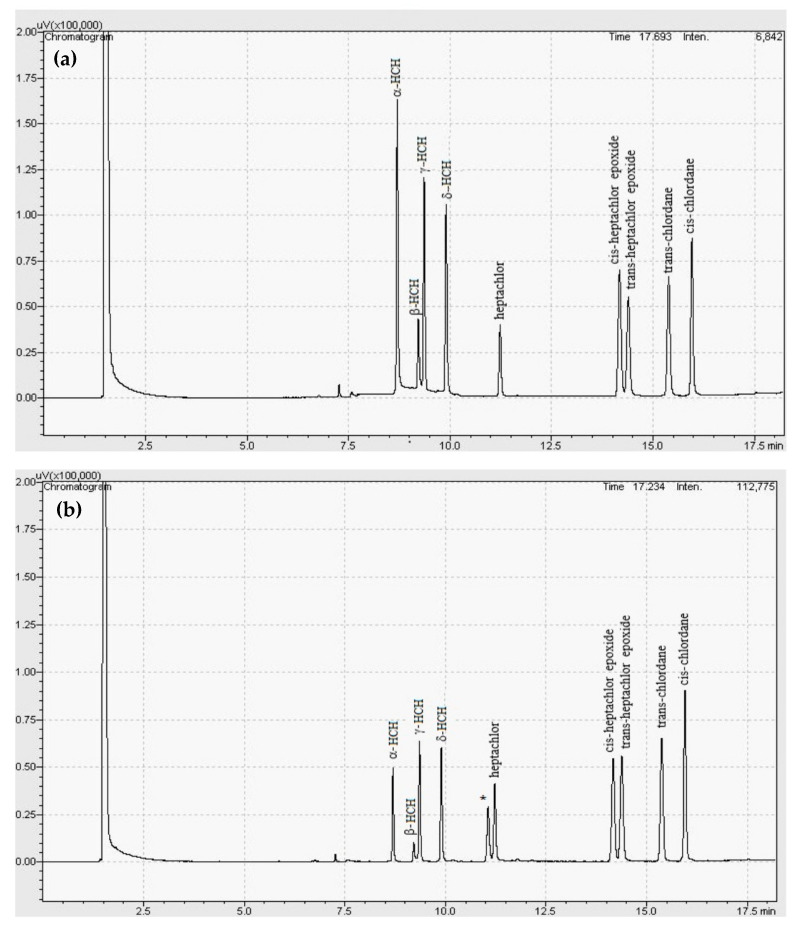
Selectivity chromatogram of the organochlorine compounds standards (**a**) 0.1 µg mL^−1^ in ACN and (**b**) 100 µg kg^−1^ in soil matrix. * Indicates some contaminant non-identified from the soil matrix.

The method was modulated to optimize chromatographic analysis time and to define the best parameters for quantification of α, β, γ, δ-HCH, heptachlor, *cis* and *trans*-heptachlor epoxide and *cis* and *trans*-chlordane organochlorines from soil samples. For that, some parameters such as the gradients of temperature were tested as follows: (1) Temperature (T) was set to 60 °C for 1 min, and then, T was increased to 180 °C at 23 °C min^−1^ for 6 min, and finally, T was increased to 330 °C at 35 °C min^−1^ for 1 min, and the speed of column gas carrier was 1.7 mL min^−1^; (2) T was set to 80 °C for 1 min, and then, T was increased to 210 °C at 25 °C min^−1^ for 6 min. After that, T was increased to 330 °C at 35 °C min^−1^ for 1 min, and the speed of column gas carrier was 1.5 mL min^−1^; (3) T was set to 90 °C for 1 min, then T was increased to 170 °C at 35 °C min^−1^ for 5 min. Posteriorly, T was increased to 250 °C at 40 °C min^−1^ for 1 min, and the speed of column gas carrier was 1.6 mL min^−1^; (4) T was set to 80 °C for 1 min, and then, T was increased to 210 °C at 25 °C min^−1^ for 7 min. T was increased to 290 °C at 35 °C min^−1^ for 1 min, and the speed of the column gas carrier was 1.7 mL min^−1^ (data not shown). The best performance was found for method 4, and its chromatogram is shown in Figure 3a.

### 3.2. Soil Classification

The organic matter content of the soil was evaluated and resulted in 0.8%, showing that the soil presented low organic content and the matrix was not likely to retain organic contaminants, which allowed its leaching to deep levels. The granulometry assay showed a balance in the soil profile, in other words, the soil presented 37% of clay, 31% of silt and 32% of sand, indicating that the soil presented characteristic grain size of clay, silty and sandy soil in similar proportions.

### 3.3. Validation

**Figure 4:**
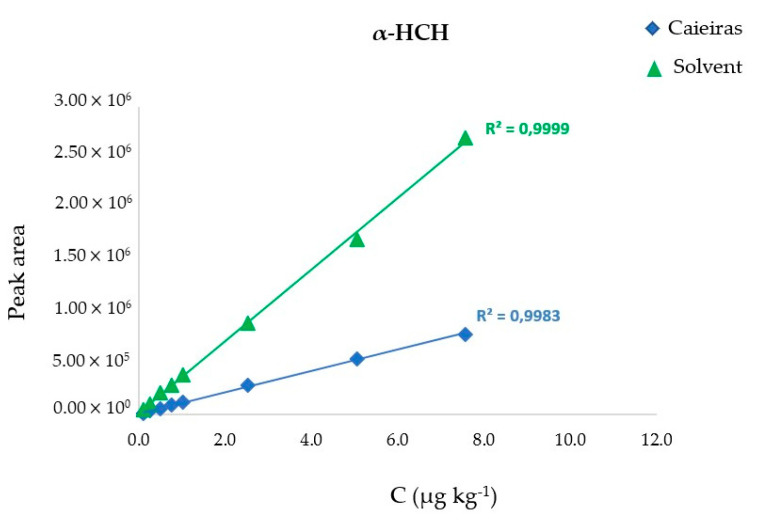
Analytical curves of α-HCH in ACN solvent and soil matrix.

Linearity was expressed through the linear regression equation and determination coefficients, which were greater than 0.991 for all compounds in both solvent and matrix groups as observed in Table 3. The linearity and selectivity are also verified in Figure 4, which illustrates the analytical curves of α-HCH in solvent and soil matrix.

Selectivity and sensitivity were observed in the chromatograms of the standard solution in solvent and soil matrix, as observed in Figure 3a,b, in which the presence of nine organochlorines at 1.0 µg kg^−1^ was observed. By observing the chromatograms in which the standard solutions were prepared at the same concentration, it was possible to note the higher intensity in the peaks of compounds 1–4 in the solvent, which might mean that the matrix presented a suppression effect over the signals of such analytes. It was possible to conclude that the signal intensities of compounds 5–9 were the same when assessed in the matrix, revealing a very small matrix effect, and thus evidencing that the matrix affects differently the evaluated analytes. Selectivity was also evaluated by F and t-tests. The results are presented in Table 4. 

Precision was evaluated in terms of variation coefficient, repeatability and reproducibility. The variation coefficient was expressed in percentage, and three concentration levels were considered, and all variation coefficients were lower than 20% as observed in Table 5.

The limit of repeatability (r) and reproducibility (R) values, as well as the concentration levels and the relative standard deviations are shown in the Table 6, and both results of repeatability and reproducibility were satisfactory according to the literature [27].

Trueness was evaluated through the Z-score test. Limits of detection and quantification were determined by the analytical curve parameters with 95–99% of assurance, analyte concentration higher than zero, and recovery, determined by two soil fortification assays, using concentrations of 1 and 2 µg g^−1^. The previously mentioned results are shown in Table 7.

### 3.4. Sample Analysis

**Figure 5:**
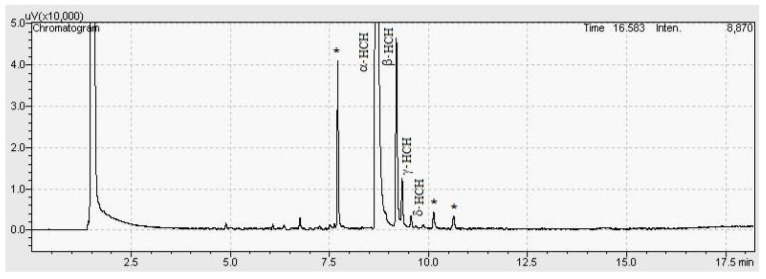
Chromatogram of the sample n1. Asterisks indicate some non-identified compounds.

**Figure 6:**
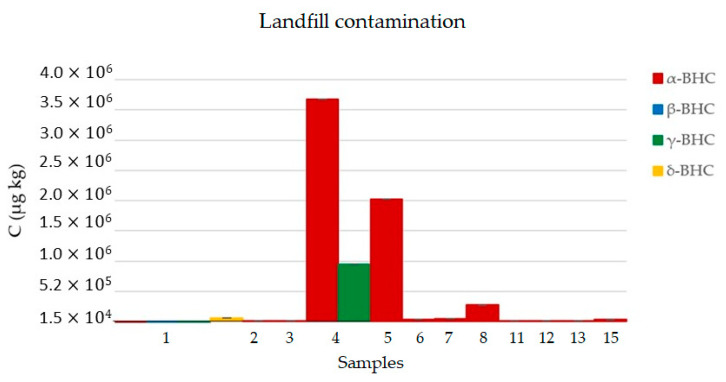
Caieiras landfill contamination results.

Sample 1 (n1) presented contamination for all HCH isomers, and the chromatogram is shown in Figure 5. Furthermore, twelve of the fifteen samples (n) presented some contamination of HCH isomers as shown in Table 8 and Figure 6. The highest concentration was found in n4 with 3.69 × 10^6^ ± 0.17 µg kg^−1^ of α-HCH and 9.7 × 10^5^ ± 0.05 µg kg^−1^ of β-HCH. The HCH isomers were quantified in other samples in the range of 1.75 × 10^4^ to 2.04 × 10^6^ µg kg^−1^, mainly by α-HCH, showing high contamination levels considering the maximum acceptable limits for residential and industrial soil according to the EPA, established as 86 and 360 µg kg^−1^ for α-HCH, 300 and 1.3 × 10^3^ µg kg^−1^ for β-HCH, 570 and 2.5 × 10^3^ µg kg^−1^ for γ-HCH. There is no established limit for δ-HCH [29].

## 4. Discussion

### 4.1. Soil Classification

The method proposed yielded optimum separation and signal of the nine organochlorines including all isomers within 18 min. Concerning the soil characterization, Caldas and colleagues evaluated the soil samples from a rice field and found a soil with low organic matter content of 0.6%, clay content of 16% and pH of 5.2 [8]. Correia-Sá and colleagues tested soil samples from 14 different places through the determination of total organic carbon, classifying the soil samples as of low or high organic matter content based on higher or lower than 2.3% organic carbon content. Eight of them presented organic matter content higher than 2.3%. The soil trace elements composition was determined, and most of the soil samples presented were predominantly siliceous, mostly as aluminum silicate, but quartz forms were also found marking the sandy and clayish profile of the soils analyzed [10].

Quinete and collaborators developed an analytical method to quantify some organochlorine pesticides in water and soil to monitor the impact of the agriculture activities expansion in the near border of Atlantic Rain Forest situated in southeastern Brazil. One of the soil samples was classified as Udorthent Eutrophic, predominantly sandy, and the other as Chernosol Argiluvic Orthic and Ultisol Dystrophic soil, mostly clayey. They found soil samples with pH in the range of 3.87 to 4.50, which is considered high acidity for soil [24].

According to the granulometric assay, the soil analyzed in this study presented contents of 31% silt, 37% clay and 32% sand, a balanced and different profile in comparison with the above-mentioned studies. A very low organic matter content of 0.8% was identified and a pH of 3.5, which is considered extremely acid for soil and tends to facilitate leaching, similarly as reported by Quinete and collaborators. Thus, considering the viability of applying the method for the evaluation of soil matrices from different places, the soil characterization parameters should be considered due to the impact of the matrix characteristics on the analytical response. Therefore, soil characterization is essential for understanding the ordinary interactions between the matrix and the contaminant, and ultimately, to determine the effects of the matrix over the quantification.

### 4.2. Method Performance and Validation 

Due to the complexity of soil analyzed, the effects of the matrix could potentially influence the chromatographic response and, consequently, the analyte quantification. In this context, such effect was studied to ensure reliability in the analytical results by analyzing the organochlorines in ACN and in the soil matrix extract. Table 3 shows linear regression equation for both solvent and matrix groups of samples. The pairs presented completely different equations indicating a different profile in the analytical curves as illustrated in Figure 4 with the analytical curve of α-HCH in solvent and in the soil matrix extract, which showed a significant difference in the slope. The linear regression equation and determination coefficient (r^2^) obtained for the solvent and matrix assays also revealed that the analytical curves of this study held optimum linearity with r^2^ higher than 0.991 for all compounds from both groups. The determination coefficient is frequently used to indicate linear fitting among the assayed points in which values higher than 0.81 are required to assure adequate linearity of the system. 

Concerning selectivity and sensitivity, the nine compounds were perfectly-identified and separated, including the impurities originated from the soil matrix. The Figure 3b shows the peaks corresponding to the pesticides in soil matrix, and a non-identified peak featuring retention times between 10.8 and 11.2 minutes, possibly related to some contaminant present in the soil matrix. Thus, the selectivity and sensitivity were considered reliable due to the adequate separation among the peaks and their intensity, comparing peak intensities of both sample groups (Figure 3a,b).

Precision is defined as the dispersion of results among independent assays, repeated from a single sample, similar sample or standards under determined conditions. The precision was assessed through the variation coefficient (VC) of three different concentration levels for all compounds at each concentration assayed (Table 5). The VC was lower than 20% for all compounds as 7 compounds presented VC lower than 5% for the concentration of 250 µg kg^−1^, and all of them presented VC lower than 5% for concentrations of 500 and 1000 µg kg^−1^, which is considered an optimum performance for multi-compound analytical methods, mainly concerning those which present a complex matrix. With regards to repeatability and reproducibility results (Table 6), the averages of the standard deviations of the seven replicates were lower than the limits of repeatability and reproducibility calculated for all compounds, and the differences between the absolute values of all replicates were lower than the limits established for each compound. Trueness was verified through the Z-score test (Table 7) and the results were lower than 2, considered satisfactory, and showed that the analytical method was exactly according to the validation guides [26,27].

Concerning the matrix effect, Fernandes and collaborators (2013), as an example, observed the matrix effect over 13 pesticides such as α, β-HCH, HCB, endrin, o, p’-DDT, among others [9]. In terms of the analytical method, the matrix tended to affect the limits of detection and quantification. Correia-Sá et al., 2012, as a further example, assessed 14 different types of soil from Portuguese regions with a wide range of soil composition and obtained different values for the limits of detection and quantification for soils of high and low organic matter content. The results for the soil of high organic matter content were 3.42 to 23.77 µg kg^−1^ and 11.41 to 79.23 µg kg^−1^, respectfully. For the soil of low organic matter content, the results were 6.11 to 14.78 µg kg^−1^ and 20.37 to 49.27 µg kg^−1^. In this study, the ranges obtained for limits of quantification and detection were 7.9 to 14.3 µg kg^−1^ and 0.3 to 3.8 µg kg^−1^, respectively [10]. 

Concerning the matrix effect in the present study, normally the matrix presents a signal increment, but in some cases, there is a reduction, as observed for compounds α, β, γ and δ-HCH in Figure 3b. Such phenomenon was likely to occur due to the soil complexity. Generally, the presence of high amounts of components in the matrix might protect the analyte from adsorption or degradation during the sample evaporation in the inlet device of the gas chromatograph, affecting substantially the response. By comparing results obtained for limits of detection and quantification of samples in both solvent and soil matrix groups (Table 7), the matrix group presented higher limits due to the signal suppression promoted by the matrix on the analytes. Otherwise. the results were numerically very similar.

As for the recovery assay, Caldas and colleagues found results between 70.3% and 120% and relative standard deviation (RSD) lower than 18.2% [24]. Fernandes and collaborators developed a multiclass pesticide residue method to determine 36 pesticides in soil from organic farming and integrated pest management areas by GC-MS/MS and QuEChERS method extraction. Mean recoveries of pesticides at each of the four concentration levels assessed between 10 to 300 µg kg^−1^ of soil ranged from 70% to 120% with RSD < 15% [9]. Correia-Sá and collaborators developed a multi-residue method to determine 14 organochlorines in 14 different types of soil in the Portuguese area using GC-ECD and extraction by optimized QuEChERS method. The recovery results were between 70% and 120% with RSD of ≤16% [10]. Rissato and colleagues developed a multi-residue method to determine some persistent organic pollutants (POPs) such as dichlorodiphenyltrichloroethane (DDT) and its metabolites, hexachlorocyclohexane (HCH) isomers and congeners of polychlorinated biphenyls in three different regions of the northeast of São Paulo, Brazil, via gas GC-MS and extraction via Soxhlet with extraction step of three hours. The samples were collected close to industrial and agricultural practice regions. Compounds such as α, β, γ, δ-HCH and heptachlor epoxide presented recoveries of 105, 85, 99, 75 and 125, respectively [34].

With respect to the present study, almost all compounds presented results between 65% and 105% (Table 7), which are optimum results considering the complexity of the matrix. Although recovery values outside the acceptable range (50–120% for complex matrix) were observed for α-HCH and γ-HCH, one must consider that the soil used to perform the validation was the original contaminated soil, and the matrix presented a considerable impact on the detection of the analytes. 

Additionally, in a multiscreen method, some compounds usually present more or less detectability by the method at a given concentration range. As an example, α-HCH presented recovery of 92.1% at soil fortification of 1.0 µg g^−1^ and of 44.2% at soil fortification of 2.0 µg g^−1^. These variations might take place due to the nature of the soil matrix and the high matrix impact on the analyte at lower concentrations. On the other hand, the isomer γ-HCH presented recovery of 211.7% at soil fortification of 2.0 µg g^−1^, which might be attributed to the matrix increment, while it presented 88.6% recovery at soil fortification of 1.0 µg g^−1^. Additionally, many other validation criteria were assessed in the present study, and the results pointed out that the method is reliable for organochlorine determination under the specified conditions. Concerning lower concentrations for the recovery test, initially, the concentrations of 0.25, 0.5 and 0.75 µg g^−1^ were evaluated, but the results were not reliable, presenting a high variation coefficient due to the contamination promoted by the spike being lower than the contamination already present in the samples, as the blank soil adopted was collected in the region and showed some contamination level that was previously determined and subtracted in the calculation. 

### 4.3. Soil Contamination Study

Comprising the contamination study, in the last decades, there has been an increasing number of studies regarding the determination of contaminated areas as well as an increase in research related to the development and optimization of analytical methodologies to assess different kinds of matrixes. Rissato and collaborators determined α-HCH in the range of 0.06 to 0.26 µg kg^−1^, 0.15 µg kg^−1^ of β and γ-HCH, 0.07 µg kg^−1^ of δ-HCH and 0.05 µg kg^−1^ of heptachlor epoxide in soil samples from three different regions of northeast São Paulo, Brazil [34]. Nearby, Quinete and colleagues found around 31 µg kg^−1^ of γ-HCH in soil samples in Rio de Janeiro, Brazil [24]. 

Finally, tracing an international comparative for pesticides in soil studies, Fernandes and colleagues determined 19 µg kg^−1^ of γ-HCH in integrated pest management soil and 15 µg kg^−1^ of the same compound in organic farming soil, both from Portugal [9]. Barron and collaborators determined organochlorines in soil and food samples from some landfill areas and family farm villages of a rural area from Tajikistan. They found higher levels in landfill areas (0.15–1.5 µg kg^−1^) than in a family farm village (0.01–0.35 µg kg^−1^) [35]. Wong and collaborators performed a comparative urban versus rural study in Canada and the UK. Among other pesticides, they quantified concentrations that were amongst the highest reported for agricultural soils on that study. They reported α+γ-HCH as chlordane, which ranged from 0.11 to 0.98 µg kg^−1^, with the highest level found at the urban site—Riverdale (0.98 µg kg^−1^), followed by the rural site—Borden (0.38 µg kg^−1^), the suburban sites—Aurora (0.11 µg kg^−1^) and North York (0.19 µg kg^−1^) in Canada [36]. 

By comparing results from the present study (Table 8) and others performed in other regions as mentioned previously, Caieiras landfill presented a higher contamination level of HCH isomers. In some sampling points, it presented a contamination level at least forty times higher than other evaluated areas, which might be explained because Caieiras landfill was an area where some pesticides were illegally and inappropriately disposed, while other studies evaluated other kinds of areas, such as Forests and agriculture fields, in which the pesticide is normally diluted prior to use.

Based on the presented data from different places around the world, the contamination quantified in the present study was found in higher levels if compared to samples analyzed by other studies in different regions, indicating a strong need for remediation, especially considering the exposure of the surrounding population. Concerning the distribution of soil contamination, n4 and n5 presented the highest levels of HCH isomers, and such sampling points were collected inside the borders of the landfill, followed by n8, which was situated outside the landfill limits. HCH isomers were found in twelve of the total fifteen samples at higher levels than the limits established by EPA for residential and industrial soil as shown in Table 1. Although the result of n14 was under the limit of quantification of the method, n15 presented an expressive contamination level and was located very close to the community area. Sampling points n9, n10, n11 and n12 are situated in commercial/industrial area with total people and vehicle access and, also presented higher contamination levels than the limits recommended by the EPA [29].

## 5. Conclusions

The multi-residue method developed presented adequate performance according to the national and international guides for validation of analytical methods. GC-ECD technique showed great performance to quantify the compounds α, β, γ, δ-HCH, heptachlor, *cis* and *trans*-heptachlor epoxide and *cis* and *trans*-chlordane. The linearity and sensitivity were evaluated through the analytical curve assay whereby the method was considered adequate based on the regression coefficients above than 0.99127 and the working range within the limits established by EPA, thus validating the method for the application proposed. The method presented adequate selectivity, highlighting its capacity of separating the compounds in the study even in the presence of isomers, which are compounds of hard separation. The precision was evaluated through the variation coefficient data, and all results were lower than 20%, indicating adequate precision. Trueness was assessed by Z-score test in which all results were lower than 2 and thus being in accordance with the established criteria. The limits of detection and quantification were lower than the maximum acceptable limits for organochlorine contaminants residues in soil according to EPA evidencing adequate sensitivity and determination capacity. The recovery was measured in the range of 65% to 105% for almost every compound and was considered acceptable for the evaluation of complex matrixes. The technique was particularly interesting considering its easy and quick performance, as well as its low cost and low reagent amounts, which makes its use environmentally justifiable.

The study enabled the application of the method to organochlorine extraction in soil matrix with suitable accuracy and contributed to Brazilian input to the Stockholm treaty, in terms of the international commitment to identify and quantify contamination and soil remediation when applicable. Moreover, the evaluation of an unstudied landfill area where considerable amounts of pesticides were illegally discarded and which is currently surrounded by a community is of interest to public health, and its economic aspects are of relevance to society. 

## Figures and Tables

**Table 1:** Maximum recommended limits for the organochlorines in residential and industrial soils (adapted from the Environmental Protection Agency—EPA) [29].

Compound	Residential Soil (µg kg^−1^)	Industrial Soil (µg kg^−1^)
α-HCH	86.0	360.0
β-HCH	300.0	1.3 × 10^3^
γ-HCH	570.0	2.5 × 10^3^
δ-HCH	-	-
Heptachlor	130.0	630.0
*Cis/trans*-heptachlor	7.0	330.0
*Cis/trans-*Chlordane	1.77 × 10^3^	7.70 × 10^3^

**Table 2:** Global positional system (GPS) coordinates of sampling at Caieiras landfill, SP, Brazil.

N	South	West	N	South	West	N	South	West
1	23°20′438″	46°49′13″	6	23°20′437″	46°49′991″	11	23°20′491″	46°49′0.87″
2	23°20′434″	46°49′132″	7	23°20′441″	46°49′441″	12	23°20′445″	46°49′105″
3	23°20′418″	46°49′3″	8	23°20′442″	46°49′977″	13	23°20′435″	46°49′113″
4	23°20′422″	46°49′3″	9	23°20′465″	46°49′0.28″	14	23°20′387″	46°49′0.56″
5	23°20′403″	46°49′988″	10	23°20′470″	46°49′470″	15	23°20′398″	46°49′0.20″

**Table 3:** Linear range, linear regression equation, and determination coefficient of solvent and matrix sample groups.

Compound	Linear Range (µg kg^−1^)	Solvent		Matrix	
		Linear Regression Equation	r^2^	Linear Regression Equation	r^2^
α-HCH	101–1010	y = 3.72 × 10^5^x + 9.86 × 10^3^	0.998	y = 1.21 × 105x − 1.651	0.999
β-HCH	103–1030	y = 91,966x – 65,633	0.999	y = 25,827x − 277.1	0.999
γ-HCH	102–1020	y = 35,554x + 874.03	0.997	y = 16,445x − 9228	0.997
δ-HCH	100–1000	y = 278,039x + 10,590	0.997	y = 18,069x −2448	0.999
Heptachlor	101–1010	y = 129,344x − 1946	0.999	y = 12,245x − 197.7	0.999
*Cis*-heptachlor epoxide	111–1114	y = 312,320x − 6795.2	0.998	y = 25,022x − 2840	0.997
*Trans*-heptachlor epoxide	105–1050	y = 232,739x − 2407	0.998	y = 271,470x − 1393	0.998
*Cis*-chlordane	116–1160	y = 286,709x + 1111.2	0.999	y = 290,550x − 1090	0.999
*Trans*-chlordane	108–1080	y = 260,109x − 2265	0.997	y = 33,889x − 4037	0.992

**Table 4:** Selectivity results obtained from F and t-tests (F tabulated equal to 4.28 and tabulated t equal to 2.179).

C (µg kg^−1^)	0.10	0.25	0.50	0.75	1.00	2.50	5.00	10.00
				**α-HCH**				
s^2^ (ACN)	3.82 × 10^6^	1.65 × 10^7^	4.28 × 10^7^	3.41 × 10^8^	1.64 × 10^9^	3.19 × 10^9^	7.93 × 10^10^	4.77 × 10^1^
s^2^ (C)	3.56 × 10^5^	2.89 × 10^6^	3.82 × 10^6^	2.04 × 10^7^	4.24 × 10^7^	2.37 × 10^7^	2.10 × 10^8^	7.51 × 10^8^
Fcalc	0.09	0.18	0.09	0.06	0.03	0.01	0.00	0.02
tcalc	40.16	47.22	58.89	26.76	17.28	28.27	10.93	23.08
				**β-HCH**				
s^2^ (ACN)	1.42 × 10^6^	2.92 × 10^7^	1.33 × 10^7^	4.09 × 10^7^	1.99 × 10^8^	1.94 × 10^8^	6.41 × 10^9^	5.15 × 10^9^
s^2^ (C)	3.38 × 10^4^	9.19 × 10^5^	1.05 × 10^6^	1.51 × 106	1.70 × 10^6^	7.06 × 10^6^	2.36 × 10^7^	6.34 × 10^7^
Fcalc	0.02	0.03	0.08	0.04	0.01	0.04	0.00	0.01
Tcalc	16.50	7.80	25.13	19.33	13.06	31.04	11.53	22.38
				**γ-HCH**				
s^2^ (ACN)	1.89 × 10^7^	7.94 × 10^7^	8.76 × 10^7^	2.48 × 10^8^	9.62 × 10^8^	1.78 × 10^9^	5.34 × 10^10^	3.47 × 10^10^
s^2^ (C)	9.80 × 10^5^	4.36 × 10^7^	8.40 × 10^6^	5.17 × 10^7^	4.07 × 10^7^	6.91 × 10^7^	2.89 × 10^8^	1.32 × 10^9^
Fcalc	0.05	0.55	0.10	0.21	0.04	0.04	0.01	0.04
Tcalc	15.32	9.53	22.83	13.50	11.27	19.39	7.45	16.60
				**δ-HCH**				
s^2^ (ACN)	4.32 × 10^6^	2.31 × 10^7^	2.42 × 10^7^	1.81 × 10^8^	9.63 × 10^8^	2.90 × 10^9^	5.96 × 10^10^	3.88 × 10^10^
s^2^ (C)	2.91 × 10^5^	1.28 × 10^7^	1.35 × 10^7^	8.69 × 10^7^	8.46 × 10^7^	1.97 × 10^7^	4.53 × 10^8^	1.18 × 10^9^
Fcalc	0.07	0.55	0.56	0.48	0.09	0.01	0.01	0.03
Tcalc	19.52	17.45	29.76	12.99	9.73	15.07	6.97	16.08
				**Heptachlor**				
s^2^ (ACN)	3.73 × 10^5^	1.95 × 10^5^	6.45 × 10^6^	2.82 × 10^7^	1.40 × 10^8^	5.22 × 10^8^	1.33 × 10^10^	9.21 × 10^9^
s^2^ (C)	3.55 × 10^5^	5.03 × 10^6^	7.46 × 10^6^	2.30 × 10^7^	5.92 × 10^7^	2.22 × 10^7^	2.25 × 10^8^	4.36 × 10^8^
Fcalc	0.95	25.85	1.16	0.82	0.42	0.04	0.02	0.05
tcalc	6.00	6.77	7.36	6.70	2.27	0.87	1.43	6.53
			***Cis*-heptachlor epoxide**					
s^2^ (ACN)	2.27 × 10^6^	9.37 × 10^6^	6.48 × 10^7^	8.10 × 10^7^	1.14 × 10^9^	1.88 × 10^9^	5.09 × 1010	3.21 × 10^10^
s^2^ (C)	7.89 × 10^5^	1.04 × 10^7^	1.03 × 10^7^	7.23 × 10^7^	1.41 × 10^8^	5.21 × 10^7^	9.18 × 10^8^	2.49 × 10^9^
Fcalc	0.35	1.12	0.16	0.89	0.12	0.03	0.02	0.08
Tcalc	7.43	5.38	8.83	4.94	4.09	8.44	4.45	11.71
			***Trans*-heptachlor epoxide**					
s^2^ (ACN)	1.14 × 10^6^	4.71 × 10^6^	5.26 × 10^7^	4.71 × 10^7^	8.10 × 10^8^	1.30 × 10^9^	3.75 × 10^10^	2.36 × 10^10^
s^2^ (C)	6.63 × 10^5^	1.52 × 10^7^	9.52 × 10^6^	9.10 × 10^7^	1.58 × 10^8^	6.44 × 10^7^	1.15 × 10^9^	2.59 × 10^9^
Fcalc	0.58	3.23	0.18	1.93	0.20	0.05	0.03	0.11
Tcalc	8.05	8.93	7.02	10.03	2.51	3.34	0.64	5.30
***Cis*-chlordane**								
s^2^ (ACN)	1.54 × 10^6^	5.88 × 10^6^	3.93 × 10^7^	1.10 × 10^8^	1.24 × 10^9^	1.96 × 10^9^	6.11 × 10^10^	2.98 × 10^1^
s^2^ (C)	3.04 × 10^5^	2.16 × 10^7^	2.62 × 10^7^	2.27 × 10^8^	3.69 × 10^8^	2.28 × 10^8^	2.82 × 10^9^	8.45 × 10^9^
Fcalc	0.20	3.67	0.67	2.07	0.30	0.12	0.05	0.28
tcalc	0.44	10.42	11.22	9.29	2.22	3.12	0.35	6.56
***Trans*-chlordane**								
s^2^ (ACN)	1.50 × 10^6^	9.31 × 10^6^	4.42 × 10^7^	7.21 × 10^7^	1.14 × 10^9^	2.28 × 10^9^	7.13 × 10^10^	4.44 × 10^10^
s^2^ (C)	3.67 × 10^5^	2.08 × 10^7^	2.41 × 10^7^	1.54 × 10^8^	2.27 × 10^8^	1.59 × 10^8^	2.16 × 10^9^	5.08 × 10^9^
Fcalc	0.25	2.23	0.54	2.14	0.20	0.07	0.03	0.11
tcalc	7.07	6.49	5.91	6.90	1.42	0.56	1.51	7.64

s^2^: standard deviation of the differences of the replicates; Fcalc: calculated F; tcalc: calculated t; (ACN): acetonitrile; (C): Caieiras.

**Table 5:** Variation coefficients (VC) for nine organochlorines in three concentration levels, 250 (C_1_), 500 (C_2_), and 1000 µg kg^−1^ (C_3_).

Compound	C_1_ (µg kg^−1^)	VC_1_ (%)	C_2_ (µg kg^−1^)	VC_2_ (%)	C_3_ (µg kg^−1^)	VC_3_ (%)
α-HCH	253	4.15	505	2.20	1010	2.66
β-HCH	258	5.64	515	4.19	1030	0.68
γ-HCH	255	1.35	510	2.53	1020	0.32
δ-HCH	250	5.08	500	0.54	1000	3.82
Heptachlor	253	2.76	505	1.77	1010	3.62
*Cis*-heptachlor epoxide	278	1.91	557	1.36	1110	1.70
*Trans-*heptachlor epoxide	263	1.11	525	0.92	1050	1.95
*Cis*-chlordane	270	2.11	540	0.56	1080	1.48
*Trans*-chlordane	290	1.98	580	1.85	1160	1.95

**Table 6:** Limit of repeatability (r), reproducibility (R) and replicates’ standard deviation averages ($\bar{S}$:) calculated for three concentration levels.

Compounds	µg mL^−1^	$\bar{S}$ Replicates	r	$\bar{S}$ Replicates	R
	0.101	0.0048	0.0134	0.0018	0.0051
α-HCH	0.505	0.0157	0.0439	0.0096	0.0269
	2.525	0.0391	0.1094	0.0218	0.0609
	0.103	0.773	5.150	0.0020	0.0056
β-HCH	0.0071	0.0472	0.1869	0.0188	0.0526
	0.0198	0.1323	0.5234	0.0879	0.2460
	0.102	0.765	5.100	0.0052	0.0144
γ-HCH	0.0071	0.0472	0.1869	0.0273	0.0763
	0.0198	0.1323	0.5234	0.0295	0.0826
	0.100	0.750	5.000	0.0010	0.0028
δ-HCH	0.0071	0.0472	0.1869	0.0215	0.0603
	0.0198	0.1323	0.5234	0.0321	0.0899
	0.101	0.758	5.050	0.0017	0.0047
Heptachlor	0.0071	0.0472	0.1869	0.0188	0.0526
	0.0198	0.1323	0.5234	0.0310	0.0867
	0.111	0.835	5.568	0.0019	0.0054
*Cis*-heptachlor epoxide	0.0071	0.0472	0.1869	0.0107	0.0299
	0.0198	0.1323	0.5234	0.0299	0.0836
	0.105	0.788	5.251	0.0019	0.0052
*Trans*-heptachlor epoxide	0.0071	0.0472	0.1869	0.0112	0.0313
	0.0198	0.1323	0.5234	0.0307	0.0861
	0.108	0.270	0.540	0.0019	0.0052
*Cis*-chlordane	0.0071	0.0472	0.1869	0.0112	0.0313
	0.0198	0.1323	0.5234	0.0307	0.0861
	0.116	0.870	5.799	0.0009	0.0027
*Trans*-chlordane	0.0071	0.0472	0.1869	0.0132	0.0369
	0.0198	0.1323	0.5234	0.0388	0.1085

**Table 7:** Results of the score test (Z). Limits of detection (LD) and quantification (LQ) for solvent and matrix groups and recovery at soil fortification of 1.0 (Rec_1_) and 2.0 µg kg^−1^ (Rec_2_).

Compound	C (µg kg^−1^)	Z	Matrix (µg kg^−1^)		ACN (µg kg^−1^)		Rec_1_	SD	Rec_2_	SD
			LD	LQ	LD	LQ	(%)	(%)	(%)	(%)
α-HCH	10.0	0.015	1.20	12.60	1.30	11.60	92.1	1.0	44.2	1.9
β-HCH	96.0	1.409	1.70	12.00	3.20	13.10	101.1	3.9	84.9	4.1
γ-HCH	233.0	0. 699	1.50	11.60	3.80	11.70	88.6	0.7	211.7	7.9
δ-HCH	101.0	0.188	0.80	11.60	1.90	12.30	77.4	4.7	69.3	1.1
Heptachlor	99.0	0.991	1.00	10.80	1.20	12.40	100.5	1.2	65.9	2.3
Cis-heptachlor epoxide	574.0	1.818	0.90	11.50	1.20	12.60	103.2	2.6	83.4	2.9
Trans-heptachlor epoxide	111.0	1.447	0.30	11.00	1.10	13.10	84.5	4.2	71.3	4.4
Cis-chlordane	281.0	1.487	0.40	7.90	1.10	12.30	105.0	4.0	84.4	3.0
Trans-chlordane	289.0	0.069	0.50	10.90	1.10	14.30	102.4	4.6	84.0	3.5

**Table 8:** Contaminant results of the samples (n) and their respective variation coefficient (VC).

n	α-HCH	β-HCH	γ-HCH	δ-HCH
C (µg kg^−1^)				
1	3.91 × 10^3^ ± 0.18	6.23 × 10^3^ ± 0.33	1.61 × 10^3^ ± 0.07	7.68 × 10^4^ ± 5.92
2	2.81 × 10^4^ ± 9.56	<LQ	<LQ	<LQ
3	2.55 × 10^4^ ± 3.28	<LQ	<LQ	<LQ
4	3.69 × 10^6^ ± 0.17	9.7 × 10^5^ ± 0.05	<LQ	<LQ
5	2.04 × 10^6^ ± 0.08	<LQ	<LQ	<LQ
6	5.67 × 10^4^ ± 4.26	<LQ	<LQ	<LQ
7	66.010 ± 4.01	<LQ	<LQ	<LQ
8	2.9 × 10^5^ ± 0.01	<LQ	<LQ	<LQ
9	<LQ	<LQ	<LQ	<LQ
10	<LQ	<LQ	<LQ	<LQ
11	1.75 × 10^4^ ± 1.25	<LQ	<LQ	<LQ
12	3.0 × 10^4^ ± 1.84	<LQ	<LQ	<LQ
13	2.55 × 10^4^ ± 1.15	<LQ	<LQ	<LQ
14	<LQ	<LQ	<LQ	<LQ
15	4.73 × 10^4^ ± 4.73	<LQ	<LQ	<LQ

LQ: Limit of quantification.
